# The role of autophagy in intracellular pathogen nutrient acquisition

**DOI:** 10.3389/fcimb.2015.00051

**Published:** 2015-06-09

**Authors:** Shaun Steele, Jason Brunton, Thomas Kawula

**Affiliations:** Department of Microbiology and Immunology, School of Medicine, University of North CarolinaChapel Hill, NC, USA

**Keywords:** autophagy, xenophagy, nutrient acquisition, intracellular pathogens, immune evasion

## Abstract

Following entry into host cells intracellular pathogens must simultaneously evade innate host defense mechanisms and acquire energy and anabolic substrates from the nutrient-limited intracellular environment. Most of the potential intracellular nutrient sources are stored within complex macromolecules that are not immediately accessible by intracellular pathogens. To obtain nutrients for proliferation, intracellular pathogens must compete with the host cell for newly-imported simple nutrients or degrade host nutrient storage structures into their constituent components (fatty acids, carbohydrates, and amino acids). It is becoming increasingly evident that intracellular pathogens have evolved a wide variety of strategies to accomplish this task. One recurrent microbial strategy is to exploit host degradative processes that break down host macromolecules into simple nutrients that the microbe can use. Herein we focus on how a subset of bacterial, viral, and eukaryotic pathogens leverage the host process of autophagy to acquire nutrients that support their growth within infected cells.

## Introduction

Food and reproduction are basic necessities for life. Intracellular pathogens infect host cells and are dependent on them for nutrients to propagate. While there is an abundance of food inside host cells, molecules are mostly sequestered in complex compounds or structures such as glycogen, lipid droplets, and proteins; forms that are not readily usable by microbial intruders. Therefore, simply gaining access to the interior of a host cell and avoiding potent innate antimicrobial host defenses is not sufficient to guarantee successful occupation and growth. Once inside, pathogens must either stimulate host cell import of metabolites or degrade intracellular storage molecules into compounds that can be transported and metabolized. There are multiple mechanisms by which intracellular pathogens accomplish this goal. For example, *Mycobacterium tuberculosis* encodes proteins to degrade host-derived lipids, such as cholesterol, for a carbon source (Griffin et al., 2012). Pathogens can also take advantage of host signaling pathways to acquire nutrients. Both *Brucella abortus* and *Salmonella enterica* thrive on the increased glucose that is imported upon activation of various peroxisome proliferation-activated receptors (PPARs) in alternatively activated monocytes (Eisele et al., 2013; Xavier et al., 2013). Recently, several pathogens have been demonstrated to exploit host cell macroautophagy for nutrients. Autophagy is a critical mechanism that host cells use to increase nutrient availability when stressed. Since infection should exert a wide range of stresses on cells, it is not surprising that a diverse range of microbes have evolved strategies to extract the products of autophagy.

Autophagy is a highly conserved, multi-faceted eukaryotic process that maintains cellular homeostasis by degrading cytosolic material. Autophagy was noted as early as 1957 during the characterization of kidney cells by transmission electron microscopy (Clark, 1957, Deter and de Duve, 1967). In 1964, autophagy was identified as a mechanism to degrade cytosolic components and mitochondria under starvation conditions (Malkoff and Buetow, 1964). Since then, autophagy has been linked to a wide range of functions including antigen presentation through major histocompatibility complex II (MHC-II), unconventional secretion of inflammatory mediators, and cell viability (Ogata et al., 2006; Munz, 2009; Dupont et al., 2011).

Autophagy is divided into several subsets based on the components being degraded. Bulk autophagy refers to non-specific cytoplasmic turnover while selective autophagy refers to autophagic degradation of specific structures. There are several distinct types of selective autophagy, which target specific cellular components such as mitochondria (mitophagy) or lipids (lipophagy). During infections, intracellular microbes are recognized, targeted, and degraded through a form of selective autophagy termed xenophagy. Although xenophagy is efficient at destroying microbes that enter the cytosol, intracellular pathogens have developed numerous evasion strategies to avoid destruction by xenophagy, including the degradation or inhibition of autophagy components, camouflaging itself in host proteins, or blocking autophagosome maturation (Table 1).

**Table 1:** **A summary of the mechanisms employed by select pathogens to induce autophagy, evade destruction through xenophagy, and pro-microbial benefits of autophagy induction**.

**Pathogen**	**Autophagy up-regulation**	**Mechanism of autophagy evasion**	**Pro-microbial effect of autophagy**	**References**
*Anaplasma phagocytophilum*	Increases autophagy via the effector ATS-1	Converts replication vacuole to modified autophagosome	Nutrient source	Niu et al., 2008, 2012
*Brucella abortus*	Likely induces via unfolded protein response (UPR)	Converts replication vacuole to modified autophagosome	Promotes subsequent infections May increase intracellular replication (controversial)	Guo et al., 2012; Starr et al., 2012; Smith et al., 2013
*Burkholderia pseudomallei*	Increases LC3 puncta formed via the bacterial effector BPSS0180	Deaminates Gln40 of ubiquitin, potentially blocks polyubiquitination TSSM may de-ubiquitinate autophagy targets *B. cenocepacia* blocks autophagosome maturation	Proposed as a nutrient acquisition mechanism (not explicitly tested)	Cui et al., 2010; Tan et al., 2010; Singh et al., 2013; Al-Khodor et al., 2014
*Chlamydia trachomatis*	Bacterial protein synthesis enhances LC3 cleavage LC3 has autophagy independent pro-bacterial effects and may not indicate increased autophagy			Beatty, 2006; Cocchiaro et al., 2008; Pachikara et al., 2009; Al-Younes et al., 2011
*Coxiella burnetti*	LC3 lipidation increases, but not p62 turnover	Converts replication vacuole to modified autophagosome	Autophagy induction enhances replication Autophagy inhibition decreases replication	Beron et al., 2002; Gutierrez et al., 2005; Newton et al., 2014; Winchell et al., 2014
*Francisella tularensis*	Increases ATG5-independent autophagy Canonical autophagy remains at basal rate	O-antigen contributes to xenophagy evasion Other factors likely involved	Nutrient source	Barel et al., 2012; Chong et al., 2012; Steele et al., 2013; Case et al., 2014
Group A *Streptococcus*	Infection increases xenophagy	SpeB degrades the autophagy adaptor molecules p62 and NRB1 Not all serotypes encode SpeB		Nakagawa et al., 2004; Barnett et al., 2013
*Legionella pneumophilia*	Irreversably inactivates LC3 with the bacterial effector RavZ to inhibit autophagy	Inhibits autophagy		Wieland et al., 2005; Price et al., 2011; Choy et al., 2012
*Listeria monocytogenes*	Llo enhances autophagy through rupture of phagosomal membrane	Camouflage via major vault protein, ARP2/3, and Ena/VASP PlcA/PlcB reduce autophagic flux		Birmingham et al., 2007; Py et al., 2007; Dortet et al., 2011; Yoshikawa et al., 2009; Tattoli et al., 2013
*Mycobacterium tuberculosis*	Targets bacteria when ESX-1 permeabilizes the phagosome Autophagy is anti-bacterial, particularly in a mouse model	Unknown, likely by remaining in a modified phagosome		Griffin et al., 2012; Watson et al., 2012
*Orientia tsutsugamuchi*	Infection induces autophagy	Unknown, but requires live bacteria		Choi et al., 2013; Ko et al., 2013
*Salmonella enterica* serovar *typhimurium*	Increases autophagy when phagosome is damaged	- SseL deubiquitinates bacterial products - Recruits autophagy components to the replicative vacuole		Mesquita et al., 2012; Tattoli et al., 2012
*Shigella flexneri*	Increases autophagy through amino acid starvation and mTOR inhibition	- IcsB through by blocking ATG5 from binding to virG - VirA suppresses autophagy		Ogawa et al., 2005; Dong et al., 2012; Tattoli et al., 2012
Chikungunya virus	Increases autophagy through ER stress and unfolded protein response (UPR)		Promotes viral replication Delays caspase-dependent cell death	Krejbich-Trotot et al., 2011; Joubert et al., 2012
Coxsackievirus	Increases LC3 cleavage, but not p62 degradation	Limits autophagosome and lysosome fusion	Enhances viral replication Autophagosomes used for replication complexes Viral exocytosis	Wong et al., 2008; Kemball et al., 2010; Robinson et al., 2014
Dengue Virus	Increases autophagy Increases lipophagy		Autophagy-derived lipids increases ATP production Maturation of infectious particles Autophagosomes used for replication complexes	Lee et al., 2008; Panyasrivanit et al., 2009; Heaton and Randall, 2010; Mateo et al., 2013
Epstein barr virus (EBV)	Rta induces autophagy through extracellular signal regulated kinase (ERK) signaling LMP1 induces autophagy, likely via UPR	Blocks autophagosome-lysosome fusion	Autophagy enhances replication Autophagosomes contribute to exocytosis	Lee and Sugden, 2008a,b Granato et al., 2014; Hung et al., 2014
Hantavirus	The glycoprotein Gn induces autophagy		Enhances replication	Hussein et al., 2012
Hepatitis B virus (HBV)	Small surface protein induces autophagy through the unfolded protein response, X protein promotes Beclin 1 translation. Observed increase in autophagosomes may be due to decreased autophagic flux	Viral X protein impairs autophagosome maturation, leading to autophagosome accumulation	Autophagosome formation enhances viral replication Autophagy contributes to HBV envelopment	Tang et al., 2009; Li et al., 2011; Liu et al., 2014
Hepatitis C virus	NS5A induces autophagy NS4B induces autophagy, likely via interactions with Rab5, Beclin 1 and VPS34	- Autophagosome maturation impaired - Impaired long-lived protein degradation	Enhances viral replication Replication does not occur in autophagosomes	Sir et al., 2008; Shrivastava et al., 2011, 2012 Mohl et al., 2012; Su et al., 2011
Herpes Simplex Virus (HSV)	ICP34.5 protein suppresses autophagy by binding to Beclin 1 US11 inhibits autophagy through PKR		An AMPK/AKT/mTOR/Beclin 1 independent form of autophagy has been proposed to enhance cell viability	Orvedahl et al., 2007; Lussignol et al., 2013; Tovilovic et al., 2013
Human Cytomegalovirus (HCMV)	Induces autophagy early independent of viral protein synthesis Inhibits autophagy late through the viral protein TRS1 interacting with Beclin 1			Yu et al., 2011; Chaumorcel et al., 2012
Human immunodeficieny virus (HIV)	Infection increases the number of autophagosomes by electron microscopy Infection results in fewer LC3 puncta and decreased Beclin 1 protein levels Discrepancy may be due to maturation defects or cell types	- Nef inhibits autophagosome maturation through an interaction with Beclin 1 - Tat inhibits autophagy in bystander cells	Autophagy enhances the number of infectious virions Autophagy processes Gag	Zhou and Spector, 2008; Kyei et al., 2009; Van Grol et al., 2010; Wang et al., 2012
Human parvovirus	Infection increases LC3 cleavage		Increased infected cell survival	Nakashima et al., 2006
Influenza A virus	Infection increases LC3 cleavage	- Matrix 2 ion channel blocks autophagosome-lysosome fusion - Matrix 2 ion channel redistributes LC3 to the plasma membrane	Increases cell survival Increases replication (controversial)	Gannage et al., 2009; Zhou et al., 2009; Beale et al., 2014
Kaposis sarcoma herpesvirus (KSHV)	Timing dependent: Viral BCL-2 binds to Beclin 1 and inhibits autophagy vFlip (K13) binds to ATG3 and prevents ATG3-LC3 interactions RTA induces autophagy during lytic cycle		Autophagy enhances lytic reactivation	Pattingre et al., 2005; Lee et al., 2009; Wen et al., 2010
Rotavirus	NSP4 leads to increased cytoplasmic calcium levels, resulting in autophagy	Blocks autophagosome maturation	Enhances viral replication	Crawford et al., 2012
*Leishmania amozonensis*	Infection increases LC3 cleavage		Enhances parasite replication	Pinheiro et al., 2009; Cyrino et al., 2012
*Toxoplasma gondii*	Autophagy increase is calcium dependent but independent of mTOR	*T*. *gondii* micronemal proteins (MICs) prevents paristoporous vacuole-lysosome fusion via activation of EGFR-Akt signaling	Enhances nutrient acquisition	Wang et al., 2009; Muniz-Feliciano et al., 2013
*Candida albicans*	Increase in LC3 cleavage			Smeekens et al., 2014
*Cryptococcus neoformans*		Autophagosomes fuse to *C. neoformans* containing vacuole, but structure has a single membrane	Enhances non-lytic exocytosis	Nicola et al., 2012

*Bacterial, viral, and eukaryotic pathogens are listed in groups. This list may not be comprehensive for what is currently known about how each pathogen interacts with autophagy*.

Several pathogens that evade xenophagic killing have incorporated autophagy into their intracellular life cycle. These microbes exploit autophagy to sustain host cell viability, increase nutrient production, and/or for non-lytic exocytosis (Table 1). Viruses also use autophagy or autophagy components for viral assembly and maturation (Table 1). In this review, we will focus on how pathogens avoid destruction by xenophagy while harvesting nutrients from autophagic degradation of host components.

## What is autophagy?

Autophagy is a constitutive process that degrades long lived-proteins, organelles, and aggregates. In mouse embryonic fibroblasts (MEFs), a common cell line used for autophagy research, the basal rate of autophagy is approximately 1–2% of the cytosolic volume of the cell (Nishida et al., 2009). A wide range of stimuli increase autophagy over the basal rate. Two major autophagy signaling nodes are the activation of the energy sensing protein AMP-activated protein kinase (AMPK) and inhibition of the mammalian target of rapamycin (mTOR). AMPK is activated in response to a low ATP to AMP ratio, such as during glucose deprivation (Carling et al., 1987; Sato et al., 1993). AMPK induces autophagy directly by phosphorylating ULK1 or indirectly through mTOR inhibition (Kim et al., 2011). mTOR is inhibited by several other stress factors besides AMPK, such as amino acid starvation or hypoxia (Jung et al., 2010).

For an in-depth review of canonical autophagy signaling, see the following reviews (Jewell et al., 2013; Galluzzi et al., 2014; Noda and Inagaki, 2015). Briefly, AMPK activation or mTOR inhibition result in ULK1 activation (Kim et al., 2011). ULK1 phosphorylates Beclin 1 and activates the kinase VPS34. ULK1 and Beclin 1–VPS34 associated complexes localize to an open, double membrane structure termed the phagophore. The phagophore is elongated by the ATG5-ATG12-ATG16L complex (Walczak and Martens, 2013). The phagophore expands to engulf cytoplasmic material while forming a double membrane vacuole termed the autophagosome. Unprocessed LC3 is cytosolic (LC3-I), but LC3 is cleaved, lipidated with phosphatidylethanolamine (LC3-II), and embedded into the autophagic membrane upon autophagy initiation (Kabeya et al., 2000). Molecules targeted for autophagic degradation are polyubiquitinated and adaptor proteins including p62, OPTINEURIN, or NDP52 bind to both LC3-II and ubiquitinated molecules (Thurston et al., 2009; Zheng et al., 2009; Wild et al., 2011). The autophagosome then fuses with a lysosome to become an autolysosome. The adaptor molecule NDP52 was recently shown to also regulate the fusion of a subset of bacteria containing autophagosomes to lysosomes by mediating binding between LC3 (which is embedded in the autophagosome), Myosin VI (a myosin motor protein that moves toward the minus end of actin) and Tom-1 (which associates with lysosomes) (Verlhac et al., 2015). The contents within the autolysosome are degraded into their components and exported to the cytosol.

Canonical autophagy is the best characterized form of autophagy, but there are several forms of non-canonical autophagy. These non-canonical forms also generate double membrane, degradative vacuoles with the same basic maturation process (phagophore to autophagosome to autolysosome). However, these non-canonical autophagosomes are initiated through different mechanisms and do not use all of the proteins or protein complexes required for canonical autophagy. One recurrent form of non-canonical autophagy associated with pathogenesis is ATG5-independent autophagy. ATG5-independent autophagy uses some of the same machinery as canonical autophagy, such as ULK1 and Beclin 1, but does not require ATG5, ATG7, or LC3 (Nishida et al., 2009). LC3 cleavage and ATG5 knockouts are commonly used to assay for xenophagy; pathogens may preferentially induce ATG5-independent autophagy to avoid xenophagy.

ATG5-independent autophagy is induced by starvation and correlates with mTOR inhibition, but mTOR inhibition alone is not sufficient to induce this form of autophagy (Nishida et al., 2009; Steele et al., 2013). ATG5-independent autophagy is critical for *Francisella tularensis* replication and the ability of *B. abortus* to infect neighboring cells (Starr et al., 2012; Steele et al., 2013). *Mycobacterium marinum* enters autophagosome-like vacuoles in an ATG5-independent manner although the function of this vacuole is unknown (Collins et al., 2009). It is unclear how ATG5-independent autophagy is preferentially induced over canonical autophagy during these infections.

## Pathogens induce xenophagy

During infections, intracellular microbes are recognized, targeted, and degraded through a form of selective autophagy termed xenophagy. Inhibition of mTOR induces xenophagy in response to extracellular or phagocytosed microbes through Toll-like recepetors (TLRs). TLRs recognize conserved microbial factors and initiate several anti-microbial processes, including xenophagy via Myd88 and Trif interacting with Beclin 1 (Delgado et al., 2008; Shi and Kehrl, 2008). Cell to cell signaling can also induce autophagy. Interferon gamma (IFN-γ) activates autophagy through IRGM1 in human cells while CD40 ligation stimulates autophagy through PI3K and Rab7; priming cells to resist microbes (Andrade et al., 2006; Singh et al., 2006).

After phagocytosis, many pathogens escape the phagosome to replicate within the cytosol. The host cell mounts a xenophagic response to the membrane damage that occurs during phagosomal escape (Tattoli et al., 2012). Once microbes reach the cytosol, they can be targeted for xenophagy through immune surveillance or by causing cell stress. Several molecules, such as Nod-1 and Nod-2, identify microbial components within the cytosol to target microbes for xenophagy. Nod-1 and Nod-2 induce xenophagy and microbial antigen processing in response to bacterial peptidoglycan (Cooney et al., 2010; Travassos et al., 2010). Microbes also induce xenophagy through a number of cell stress mechanisms. *B. abortus* secreted TcpB induces endoplasmic reticulum stress via the unfolded protein response (UPR) pathway while *Toxoplasma gondii* increases intracellular calcium levels to induce autophagy (Qin et al., 2008; Wang et al., 2009; Smith et al., 2013).

Lastly, xenophagy can also be directly induced by microbial proteins (Table 1). For example, *Shigella flexneri* exported VirG polymerizes actin to propel the bacteria through the cytosol (Makino et al., 1986). ATG5 binds to VirG and initiates autophagosome formation without upstream autophagy signaling (Ogawa et al., 2005). However, *S. flexneri* also produces IcsB which blocks ATG5 from binding to VirG, thus inhibiting xenophagy (Ogawa et al., 2005). Likewise, the viral protein NS4B in Hepatitis C virus (HCV) induces autophagy by interacting with a Rab5/Beclin 1/VPS34 complex (Su et al., 2011).

## Pathogens have evolved complex xenophagy evasion mechanisms

Xenophagy is typically extremely effective at destroying microbes that enter the cytosol. For example, some serotypes of Group A Streptococcus (GAS) invade host cells, escape into the cytosol, and are then destroyed by xenophagy (Nakagawa et al., 2004; Joubert et al., 2009). Xenophagy effectively blocks these serotypes from using the cytosol as a replicative niche. To defend themselves, most intracellular pathogens have evolved mechanisms to either inhibit or evade xenophagy (Table 1). Some GAS serotypes encode SpeB, which degrades the xenophagy adaptor proteins p62 and NRB1 (Barnett et al., 2013). GAS serotypes that are normally destroyed by xenophagy can be functionally complemented for xenophagy evasion and intracellular replication by expressing SpeB (Barnett et al., 2013).

To inhibit autophagy, pathogens frequently impair the function of xenophagy machinery. The RavZ protein secreted by *Legionella pneumophilia* inactivates LC3, effectively blocking autophagy in infected cells (Choy et al., 2012). Human Cytomegalovirus (HCMV), Herpes Simplex virus, and Kaposis sarcoma herpesvirus inactivate Beclin 1 to inhibit autophagy at specific points in their life cycle (Table 1). Many viruses, such as Coxsackievirus, Hepatitis B virus, and HIV, inhibit autophagosome-lysosome fusion, functionally inhibiting xenophagy (Table 1). The exact mechanism by which these viruses block autophagosome maturation is unknown, but many different RNA viruses encode proteins that interact with LC3, p62, NDP52, or NRB1 (Gregoire et al., 2011; Judith et al., 2013). These proteins have several roles in xenophagy, but microbes may alter autophagosome maturation by manipulating these proteins (Verlhac et al., 2015).

A few pathogens evade xenophagy without inhibiting autophagy. *Listeria monocytogenes* camouflages itself by binding to the host proteins ARP2/3, major vault protein (MVP), and ena/VASP (Yoshikawa et al., 2009; Dortet et al., 2011). Many bacterial and eukaryotic pathogens modify phagosomes and are likely hidden from xenophagy targeting by remaining within a modified vacuole. *M. tuberculosis* and *S. enterica* typically reside in modified phagosomes but bacteria that disrupt the phagosomal membrane are rapidly destroyed by xenophagy (Tattoli et al., 2012; Watson et al., 2012). Vacuolar *M. tuberculosis* and *T. gondii are* degraded via autophagy when autophagy is stimulated by external sources, such as CD40 ligation or IFN-γ (Andrade et al., 2006; Ling et al., 2006; Singh et al., 2006). Certain pathogens evade xenophagy by altering or destroying the components that target the microbes for degradation. *S*. *enterica* de-ubiquitinates aggregates with the effector protein SseL to prevent the aggregates from being degraded via autophagy (Mesquita et al., 2012). Likewise, *B. pseudomallei* encodes the de-ubiquitinase TssM which blocks several innate immune signals including the NF-kB and type 1 IFN pathways and has been proposed as a potential autophagy evasion mechanism (Tan et al., 2010; Gong et al., 2011). A few other cytosolic pathogens, such as *Orientia tsutsugamuchi* and *F. tularensis*, induce autophagy but the mechanisms of xenophagy evasion are not clear (Choi et al., 2013; Ko et al., 2013; Steele et al., 2013).

## Pathogens harvest autophagy derived nutrients for replication

Intracellular microbes acquire nutrients from a range of sources, but generally rely on macromolecule degradation or nutrient import. Most basic nutrients within cells (amino acids, fatty acids, and carbohydrates) are incorporated into macromolecules (proteins, lipid droplets, and glycogen, respectively). In uninfected cells, these macromolecules are primarily degraded by autophagy to increase the amount of basic nutrients so the cell can build new structures. Thus, autophagy can increase the intracellular pool of nutrients that pathogens can access. Microbes can divert the nutrient by-products of autophagy toward microbial replication rather than for use by the cell. Dengue virus, *F. tularensis, Anaplasma phagocytophilum*, and *T. gondii* all induce autophagy, evade autophagic degradation, and harvest the autophagy derived nutrients for replication through different mechanisms (Wang et al., 2009; Heaton and Randall, 2010; Niu et al., 2012; Steele et al., 2013). Additionally, *B. pseudomallei, Coxiella burnetii*, and *Leishmania amozonensis* have impaired replication when autophagy is inhibited and nutrient acquisition has been implicated as a potential explanation for this phenotype.

Dengue virus requires the degradation of lipid droplets via autophagy for optimal replication (Heaton and Randall, 2010). Dengue virus infections increase cellular levels of autophagy and the resulting autophagosomes form around and degrade lipid droplets. The triglycerides derived from the lipid droplets are catabolized via mitochondrial β-oxidation, generating ATP. Thus, autophagy produces energy for the cell to indirectly enhance viral replication (Heaton and Randall, 2010). In addition to energy production, Dengue virus modifies autophagosomes or amphisomes to form a replicative niche (Lee et al., 2008; Panyasrivanit et al., 2009; Mateo et al., 2013). Rather than being degraded through xenophagy, autophagy contributes to the maturation of infectious particles (Mateo et al., 2013).

*F. tularensis* replicates in the cytosol of infected cells and induces an ATG5-independent, non-canonical form of autophagy. *F. tularensis* harvests amino acids from ATG5-independent autophagy for optimal intracellular replication. The amino acids are used for protein synthesis and are also metabolized as a major carbon source. *F. tularensis* bacteria are frequently adjacent to autophagosomes (Steele et al., 2013), indicating that *F. tularensis* is in the optimal physical location to compete with the host for autophagy derived nutrients. Although *F. tularensis* bacteria are frequently adjacent to autophagosomes, live bacteria are rarely degraded by xenophagy (Chong et al., 2012; Steele et al., 2013). O-antigen contributes to *F. tularensis* xenophagy evasion, but other effectors are also likely to be involved (Case et al., 2014).

*A. phagocytophilum* replicates in a vacuolar compartment and recruits autophagosomes directly to its replicative inclusions. *A. phagocytophilum* induces autophagy with the type IV secretion system (T4SS) effector Ats-1. Ats-1 binds to Beclin 1 and induces autophagosome nucleation directly rather than signaling through mTOR. Ats-1 induced autophagosomes localize with the inclusion membrane, suggesting that autophagosomes fuse with the inclusion body so that the bacteria can acquire the by-products of autophagic degradation. Inhibition of autophagy decreases *A. phagocytophilum* replication due to amino acid deficiency (Niu et al., 2008, 2012). Likewise, *C. burnetti* induces autophagy to enhance replication (Gutierrez et al., 2005; Newton et al., 2014; Winchell et al., 2014). *C burnetti* enters cells upon phagocytosis and modifies the phagosome to form a *C. burnetti* containing vacuoles (CCV). CCVs promiscuously fuse with other CCVs, endosomes and autophagosomes using the T4SS effector Cig2 (Newton et al., 2014; Winchell et al., 2014). When autophagy is impaired, CCVs do not fuse with one another and there is a severe replication defect (Gutierrez et al., 2005; Newton et al., 2014). The autophagosomes recruited to the CCV contain LC3, p62, and LAMP-1, suggesting that the autophagosomes that are recruited to CCVs have already fused with lyosomes (Klionsky et al., 2012; Winchell et al., 2014). Since artificially enhancing autophagy further increases *C. burnetti* replication, the fusion of autophagosomes with the CCV has been postulated as a nutrient and membrane acquisition mechanism (Gutierrez et al., 2005; Winchell et al., 2014).

*T. gondii* induces autophagy in infected host cells in a calcium dependent, mTOR independent manner (Wang et al., 2009). Inhibiting autophagy decreases *T. gondii* replication and parasite replication is rescued by supplementing with additional amino acids (Wang et al., 2009). Unlike its bacterial counterparts, fusion of *T. gondii* containing parasitophorous vacuoles (PVs) with autophagosomes leads to parasite destruction (Muniz-Feliciano et al., 2013). *T. gondii* activates EGFR and AKT to inhibit PV-autophagosome fusion with EGF-MICs, primarily MIC3 and MIC6 (Muniz-Feliciano et al., 2013).

Exploiting autophagy for nutrients is a recurrent theme in the pathogenesis of a diverse range of microbes. Several other microbes have enhanced replication when autophagy is induced and impaired intracellular replication when autophagy is inhibited, such as Chikungunya virus, *B. pseudomallei* and *L. amazonensis* (Krejbich-Trotot et al., 2011; Cyrino et al., 2012; Singh et al., 2013). *B. pseudomallei* encodes the protein BPSS0180, which induces autophagy and is required for optimal intracellular replication (Singh et al., 2013). Similarly, *L. amazonensis* induces autophagy and has a replication defect when cells are deficient for autophagy (Cyrino et al., 2012). The role of autophagy in enhancing replication of these pathogens is unknown, but nutrient acquisition is a likely explanation for these phenotypes.

## Conclusions and perspectives

Autophagy has been linked to both nutrient acquisition and pathogen destruction for decades and it has recently become clear that a diverse range of pathogens harvest autophagy derived products to enhance replication (Wang et al., 2009; Heaton and Randall, 2010; Niu et al., 2012; Steele et al., 2013). Autophagy derived nutrient acquisition is relatively straightforward in bacterial pathogens. *F. tularensis* is adjacent to autophagosomes while *C. burnetii* and *A. phagocytophilum* recruit autophagosomes to their replicative vacuoles (Gutierrez et al., 2005; Niu et al., 2012; Steele et al., 2013; Newton et al., 2014). These pathogens likely harvest the autophagy derived nutrients immediately after macromolecules are degraded. *T. gondii* acquires nutrients via host cell autophagy, but it is unclear how the parasites out-compete the host for autophagy by-products (Wang et al., 2009).

In contrast, the role of autophagy in viral nutrient acquisition is less straightforward. For example, Dengue virus does not directly incorporate the autophagy by-products into structural components. Instead, autophagy increases the amount of intracellular ATP, providing energy to the cell to support viral replication (Heaton and Randall, 2010). Additionally, many viruses benefit from other facets of autophagy, such as enhanced cell viability or the maturation of infectious particles, further confounding the role of autophagy in viral nutrient acquisition (Table 1). Viruses that induce autophagy are likely to benefit from the increase in intracellular nutrients, although this benefit may be indirect, as with Dengue virus. It is important to note that autophagy still degrades host macromolecules in cells infected with microbes that block autophagy, indicating that autophagy derived nutrients are available to microbes that can outcompete the host (Sir et al., 2008). Further investigation is needed to determine if autophagy derived nutrients are incorporated into viral macromolecules.

Autophagy is one of many mechanisms that intracellular pathogens usurp to acquire nutrients. Intracellular pathogens must acquire nutrients from the host cell to survive and propagate. These pathogens can acquire nutrients either by altering eukaryotic cell metabolism to increase nutrient import or by degrading macromolecules within the host through processes such as autophagy. For example, HCMV increases the expression of GLUT4 to increase glucose import for replication while *L. pneumophila* co-opts proteosomal degradation for amino acids (Price et al., 2011; Yu et al., 2011). It is likely that intracellular pathogens exploit multiple, additive mechanisms for nutrient acquisition. *F. tularensis* requires autophagy and *L. pneumophila* requires proteosomal degradation for optimal replication, but both of these pathogens also up-regulate the expression of the host amino acid transporter SLC1A5 in macrophages which further enhances replication by increasing amino acid transport into the infected cell (Wieland et al., 2005; Barel et al., 2012). Inhibiting macromolecule degradation or import for these bacteria impairs, but does not block, replication, suggesting that these different nutrient acquisition strategies are additive. This is likely a common theme for microbial nutrient acquisition.

Research into how pathogens acquire nutrients is still in its infancy, particularly in how microbes acquire nutrients via autophagy. ATG22, Avt3, and Avt4 contribute to amino acid efflux from autophagosomes in yeast, but the mechanisms for nutrient efflux from mammalian cell autophagosomes is not well-defined (Yang et al., 2006). Other critical questions that remain largely unanswered are which nutrients microbes acquire from autophagy and the degree to which microbes rely on autophagy for nutrients. Either glucose or amino acids rescues *T. gondii* replication in autophagy deficient cells whereas *F. tularensis* and *A. phagocytophilum* primarily acquire amino acids from autophagy, but may also harvest other autophagy derived nutrients such as lipids (Wang et al., 2009; Niu et al., 2012; Steele et al., 2013). Even microbes that do not induce autophagy or actively inhibit autophagy can compete with the host for autophagy derived nutrients. Defining host cell metabolic processes that specific pathogens depend on to acquire useable nutrients could reveal new infection treatment strategies based on interfering with host processes to augment or supplant more traditional antimicrobial therapies.

### Conflict of interest statement

The authors declare that the research was conducted in the absence of any commercial or financial relationships that could be construed as a potential conflict of interest.
